# Association of Nighttime Systolic Blood Pressure With Left Atrial-Left Ventricular–Arterial Coupling in Hypertension

**DOI:** 10.3389/fcvm.2022.814756

**Published:** 2022-02-24

**Authors:** Qiaobing Sun, Yu Pan, Yixiao Zhao, Yan Liu, Yinong Jiang

**Affiliations:** ^1^Department of Cardiology, First Affiliated Hospital of Dalian Medical University, Dalian, China; ^2^Department of Geriatric Cerebrovasology, Baoji Central Hospital, Baoji, China

**Keywords:** nighttime systolic blood pressure, circadian blood pressure pattern, 2 dimensional speckle tracking echocardiography, left atrial-left ventricular-arterial coupling hypertension, left atrial stiffness

## Abstract

**Objective:**

Hypertension (HT) induces left atrial (LA) and left ventricular (LV) dysfunction, and an increase in arterial stiffness. In this study, we investigated the association between LA-LV–arterial coupling and nighttime systolic blood pressure (BP) as well as BP circadian rhythm in essential hypertension.

**Methods:**

We enrolled 290 HT patients. All subjects were evaluated by 2- dimensional speckle tracking echocardiography (2DSTE), ambulatory 24 h BP monitoring (ABPM), and brachial–ankle pulse wave velocity (PWV). According to BP patterns, these patients were divided into two groups, which included dippers (*n* = 111), patients with a >10% reduction in BP at nighttime; non-dippers (*n* = 179), patients with a <10% reduction in BP at nighttime. 2D-STE based LA and LV strains were studied and the following parameters were measured, LV global longitudinal strain (GLS), LA reservoir strain (LA_S−S_), LA conduit strain (LA_S−E_), and LA booster pump strain (LA_S−A_). LA stiffness index (LASI) defined as the ratio of E/e′ to LA_S−S_, and PWV-to-GLS ratio (PWV/GLS) were calculated to reflect LA-LV–arterial coupling. Furthermore, we also explored the correlation between LASI (or PWV/GLS) and ambulatory blood pressure indexes.

**Results:**

Left atrial stiffness index was significantly higher in non-dippers [0.29 (0.21, 0.41)] than in dippers [0.26 (0.21, 0.33)] (*P* < 0.05). PWV/GLS was significantly higher in non-dippers [−80.9 (−69.3, −101.5)] than in dippers [−74.2 (−60.2, −90.6)] (*P* < 0.05). LA_S−S_, LA_S−E_, LA_S−A_,and LV GLS were significantly lower in non-dippers than in dippers (*P* < 0.05). Multivariate linear regression analysis revealed that nighttime systolic BP was independently correlated with LASI and PWV/GLS, even adjusted for multiple clinical risk factors, LVMI, and LVEF.

**Conclusions:**

The dipping pattern of BP was related to the abnormalities of myocardial mechanics and LA-LV–arterial coupling. However, absolute nocturnal systolic BP value maybe more important than BP circadian profile in the progression of abnormal LA-LV–arterial coupling.

## Introduction

All hypertension (HT) guidelines recommend monitoring the ambulatory blood pressure (BP) in addition to assessing clinical BP (1–4). Ambulatory blood pressure monitoring (ABPM) can provide 24 h BP data comprehensively, including daytime BP, nighttime BP, and the BP circadian rhythm. Disruption of the BP circadian rhythm and nocturnal hypertension have been revealed to be an important contribution to target organ damage (TOD) and many adverse cardiovascular events in hypertension (5–12).

Left ventricle pumping blood into the arterial system depends on dynamic regulation of preload, afterload, and myocardial contractility. The optimal left ventricular (LV)–arterial coupling is important for the proper function of cardiovascular system (13). In addition to interacting with systemic arteries, left ventricle also couples with left atrium. Left atrium regulates LV filling and cardiovascular performance by its reservoir, conduit, and boost pump functions. Therefore, the left atrial (LA)-LV–arterial system interaction can be recognized as the key indicator of global cardiovascular performance. It is well known that hypertension induces LV hypertrophy and dysfunction, LA enlargement, and arterial stiffness increase. Recent study has shown that left atrial stiffness index (LASI), which is defined as the ratio of transmitral flow velocity in early diastole to mitral annular myocardial velocity (E/e′)/peak systolic LA strain, is the indicator of both LA stiffness and LA–LV coupling, and furthermore, LASI can be recognized as an early marker of TOD in hypertension (14).

Previous studies have revealed that abnormal BP circadian patterns and nocturnal hypertension are correlated with LV structural and functional remodeling (5, 6, 15), impaired LA function (16–18), and arterial stiffness (19, 20). So far, however, there is no study investigating the relationship between nocturnal hypertension as well as BP circadian rhythm and LA-LV–arterial coupling.

In this study, we aimed to evaluate LA, LV mechanics, and arterial stiffness in essential hypertensive patients. Furthermore, we investigated the correlation of nocturnal hypertension and BP circadian rhythms with LA-LV–arterial coupling and aimed to determine whether BP circadian pattern or absolute value of nighttime BP had a stronger association with LA-LV–arterial coupling.

## Methodology

### Study Population

A total of 290 essential hypertensive patients (20–75 years old) were recruited from the Hypertension and Heart Failure Ward at the Cardiac Department of the First Affiliated Hospital of Dalian Medical University from October 2018 to September 2019. According to BP patterns, these patients were divided into two groups, which included dippers (*n* = 111), patients with a >10% reduction in BP at nighttime; non-dippers (*n* = 179), patients with a <10% reduction in BP at nighttime. The exclusion criteria were as follows: secondary hypertension, systolic heart failure, moderate or severe valvular disease, history of atrial fibrillation, coronary artery disease (history of angina pectoris, myocardial infarction or more than 50% stenosis in one of coronary arteries on coronary computed angiography), chronic renal failure (eGFR <45 ml/min/1.73 m^2^), obstructive sleep apnoea, or night workers. The research proposal was approved by the Ethics Committee of the First Affiliated Hospital of Dalian Medical University. This study complied with the principles of the Declaration of Helsinki. All patients provided written informed consent before enrolment.

### Blood Pressure Measurement

All patients monitored a 24-h ambulatory BP using an oscillometric device (Spacelabs 90207; Spacelabs, Redmond, Washington, USA). Automatic BP measurement was obtained every 30 min during daytime, from 07:00 to 23:00, and every 1 h during nighttime, and from 23:00 to 07:00. Then, these recordings were analyzed to obtain the mean 24 h, daytime and nighttime systolic BP (SBP), and diastolic BP (DBP). Patients were asked to have a regular daily schedule and remain still and silent while the BP being measured. The percentage of nocturnal BP dipping was calculated using the following formula: (%) 100 × [(daytime SBP—nighttime SBP)/daytime SBP]. The BP dipping pattern was defined as a more than 10% decrease in nocturnal SBP compared with diurnal SBP, and a <10% decrease in nocturnal SBP was regarded as non-dipping pattern.

### Arterial Stiffness Assessment

Brachial–ankle pulse wave velocity (PWV) was used to evaluate arterial stiffness, which was evaluated automatically by a pulse pressure analyzer (BP-203RPEIII; OMRON HEALTH-CARE Co., Ltd., Tokyo, Japan).

### Echocardiographic Measurements

Echocardiographic measurements were performed for all patient using a Vivid E9 ultrasound system (GE Vingmed Ultrasound, Horten, Norway) equipped with an M5S phased array transducer (2.5–5.0 MHz), following the guideline of the American Society of Echocardiography (21). All echocardiographic images were saved digitally for later analysis.

The maximum LA volume (LAV) was measured immediately before mitral valve opening from apical four-chamber and two-chamber views using the biplane-modified Simpson's rule. The LAV index (LAVI) was obtained by LAV indexed to body surface area (BSA). The LV chamber dimension and wall thickness were obtained from the LV parasternal long-axis view. The LV mass (LVM) was assessed using the formula recommended by the American Society of Echocardiography (21): LVM = 0.8 ×1.04 [(LVD + IVST + LVPWT)^3^-LVD^3^] + 0.6. The LVM index (LVMI) was obtained by LVM indexed to BSA. The LV ejection fraction (LVEF) was assessed using the biplane modified Simpson's method.

### LA and LV Strain Analysis

Two-dimensional speckle tracking echocardiography (2DSTE)-derived LA strain analysis was obtained from standard 2D greyscale images of apical four-chamber and two-chamber views at a frame rate of 40–60 frames/sec. Three consecutive cardiac cycles were stored digitally for offline analysis with Echo PAC software (Vivid 9, GE Vingmed Ultrasound).

The LA endocardial surface was manually traced at end-systole using a point-and-click approach. Automated tracking of myocardial speckles was generated by the software and manually adjusted to cover the entire thickness of the LA myocardium. LA wall was divided into 6 segments automatically by the software, and longitudinal strain curve was then generated for each segment. The LA longitudinal strain (LA_S_) was required by averaging all the strains, which were obtained by all accepted segments in both apical views. LA strain was assessed using R-R gating (zero strain was set at LV end-diastole). The first positive peak of the LA strain curve was LA_S−S_ (peak atrial strain during ventricular systole), which was related to LA reservoir function. The second positive peak was LA_S−A_ (peak atrial longitudinal strain in late diastole), which was related to LA booster pump function and measured at the onset of the P-wave. LA_S−E_ (peak atrial longitudinal strain in early diastole) was defined as the difference between LA_S−S_ and LA_S−A_, which was related to LA conduit function (22). The LA stiffness index (LASI) was defined as the ratio of E/e′ to LA_S−S_, which was related to LA–LV coupling.

Global longitudinal LV strain (GLS) was obtained from standard 2D greyscale images of apical four-chamber, three-chamber, and two-chamber views (23). PWV/GLS was calculated and regarded as the indicator of LV–arterial coupling.

### Statistical Analysis

SPSS software 21.0 (SPSS, Chicago, Illinois, USA) was used for the statistical analysis. The Kolmogorov–Smirnov test was used to assess the normality of data. Continuous variables were presented as the mean ± standard deviation, and categorical variables were presented as percentages. Differences between two independent groups of normally distributed continuous variables were assessed using independent-samples *T* test, and non-normally distributed continuous variables using the Mann–Whitney U test. Differences between categorical variables were assessed using the chi-square test, and Fisher's exact test was used when appropriate. A *P* value <0.05 was considered statistically significant. Multivariate linear regression analysis was performed to evaluate the association of nighttime SBP, the percentage of nighttime SBP decline, and BP circadian rhythm with LASI and PWV/GLS. Four models were developed. Model 1 involved adjusting for age, sex, and body mass index (BMI). Model 2 included Model 1 variables plus hypertension duration and the comorbidity of diabetes. Model 3 included Model 2 variables plus glycosylated hemoglobin (HbA1c), triglyceride (TG), total cholesterol (TC), high-density lipoprotein cholesterol (HDL-C), low-density lipoprotein cholesterol (LDL-C), and eGFR. Model 4 included Model 3 plus LVMI, and LVEF. Intra-observer and inter-observer variabilities for LA strain were analyzed in 20 subjects by evaluating the intra-class correlation coefficients (ICCs). For LA reservoir, conduit, and boost pump strain, the intraclass correlation coefficients for intraobserver variability were 0.930, 0.965, and 0.907, respectively; and the intraclass correlation coefficients for the interobserver variabilities were 0.903, 0.938, and 0.867, respectively.

## Results

### Clinical Characteristics of the Study Population

Table 1 showed the clinical characteristics of the study population. Both groups were comparable in sex distribution, smoking status, the comorbidity of diabetes, hypertension duration, and BMI. Non-dippers were older than dippers (*P* < 0.05). There were no significant differences in the levels of fasting plasma glucose (FPG), HbA1c, TC, TG, LDL-C, HDL-C, B-type natriuretic peptide (BNP), and high-sensitive cardiac troponin I (hs-cTnI) between both hypertensive groups. The eGFR was within the normal range in both groups, but it was significantly lower in non-dippers than in dippers (*P* < 0.05). Antihypertensive medications were comparable in both hypertensive groups.

**Table 1:** Clinical statistics of the study population.

	**Dippers**,	**Non-dippers**,	***P***
	***n* = 111**	***n* = 179**	
Age (years)	49.0 ± 12.3	52.5 ± 12.7*	0.021
Sex (*n*, %) males	56 (50.5%)	106 (59.2%)	0.147
Smoker (%)	28 (25.2%)	63 (35.2%)	0.091
DM (%)	30 (27.0%)	58 (32.4%)	0.360
HT duration(years)	5.0 (1.6, 10.0)	5.0 (2.0, 10.0)	0.380
eGFR (ml/min/1.73 m^2^)	116.3 ± 27.3	107.4 ± 23.7	0.004
BMI (kg/m^2^)	27.0 ± 3.7	27.1 ± 3.6	0.881
FPG (mmol/L)	5.4 (4.7, 6.3)	5.2 (4.6, 6.0)	0.326
HbA1c (%)	5.9 (5.5, 6.4)	5.9 (5.6, 6.4)	0.475
TC (mmol/L)	4.6 ± 0.9	4.8 ± 1.1	0.268
TG (mmol/L)	1.5 (1.1, 2.2)	1.5 (1.0, 2.1)	0.689
LDL-C (mmol/L)	2.7 ± 0.7	2.8 ± 0.8	0.491
HDL-C (mmol/L)	1.2 ± 0.3	1.2 ± 0.3	0.757
BNP (ng/L)	16.7 (9.3, 42.2)	24.4 (11.0, 46.9)	0.234
Hs-cTnI (μg/L)	0.01 (0.01, 0.01)	0.01 (0.00, 0.01)	0.841
Medications			
CCB (%)	72 (64.9%)	115 (64.2%)	1.000
ACEI/ARB/ARNI (%)	47 (42.3%)	76 (42.5%)	1.000
β-blockers (%)	40 (36.0%)	45 (25.1%)	0.063
Diuretics (%)	11 (9.9%)	23 (12.8%)	0.574

*DM, diabetes mellitus; HT duration, hypertension duration; BMI, body mass index; FPG, fasting plasma glucose; HbA1c, glycosylated haemoglobin; TC, total cholesterol; TG, triglycerides; LDL-C, low-density lipoprotein cholesterol; HDL-C, high-density lipoprotein cholesterol; BNP, B-type natriuretic peptide; Hs-cTnI, High-sensitive cardiac troponin I; CCB, calcium antagonists; ACEI, angiotensin-converting enzyme inhibitor; ARB, angiotensin II receptor blocker; ARNI, Angiotensin receptor-neprilysin inhibitor*.

### The 24 h ABPM Parameters

The 24 h mean SBP (24 h-SBP), 24 h mean DBP (24 h-DBP), the daytime mean SBP (d-SBP), and DBP (d-DBP) were not significantly different between both groups (*P* > 0.05). However, nighttime mean SBP (n-SBP) and nighttime mean DBP (n-DBP) were significantly higher in non-dippers compared with dippers (*P* < 0.001). The percentages of n-SBP and n-DBP declines were significantly lower in non-dippers compared with dippers (*P* < 0.001) (Table 2).

**Table 2:** 24 h ABPM Parameters of the study population.

	**Dippers**,	**Non-dippers**,	***P***
	***n* = 111**	***n* = 179**	
24 h-SBP (mmHg)	138.9 ± 18.4	142.9 ± 19.5	0.079
24 h-DBP (mmHg)	87.2 ± 13.5	89.1 ± 14.4	0.276
24 h-HR (bpm)	73.1 ± 9.9	72.2 ± 10.0	0.461
d-SBP (mmHg)	144.3 ± 19.0	144.2 ± 19.6	0.956
d-DBP (mmHg)	91.0 ± 13.9	90.2 ± 14.4	0.650
d-HR (bpm)	75.7 ± 10.4	73.9 ± 10.3	0.153
*n*-SBP (mmHg)	122.5 ± 16.7	138.7 ± 20.4	<0.001
*n*-DBP (mmHg)	76.0 ± 12.9	85.4 ± 15.5	<0.001
*n*-HR (bpm)	64.1 ± 9.2	65.3 ± 9.6	0.321
Percentage of nSBP decline (%)	15.1 ± 3.2	3.8 ± 5.5	<0.001
Percentage of nDBP decline (%)	16.5 ± 5.1	5.3 ± 7.1	<0.001

*24 h-SBP, 24 h mean systolic blood pressure; 24 h-DBP, 24 h mean diastolic blood pressure; 24 h-HR, 24 h mean heart rate; d-SBP, daytime mean systolic blood pressure; d-DBP, daytime mean diastolic blood pressure; d-HR, daytime mean heart rate; n-SBP, nighttime mean systolic blood pressure; n-DBP, nighttime mean diastolic blood pressure; n-HR, nighttime mean heart rate*.

### Conventional Echocardiographic Parameters

The LAVI, E/A ratio, and E/e′ ratio were not significantly different between both groups (*P* > 0.05). The LVMI was significantly higher in non-dippers than in dippers (*P* < 0.05). Compared with dippers, LVEF was statistically significantly lower in non-dippers (*P* < 0.05), even though LVEF of both groups were within normal range (Table 3).

**Table 3:** Conventional echocardiographic parameters.

	**Dippers**,	**Non-dippers**,	***P***
	***n* = 111**	***n* = 179**	
LAVI (mL/m^2^)	26.3 ± 7.3	27.6 ± 7.9	0.110
LVMI (g/m^2^)	98.5 ± 23.7	108.4 ± 28.7	0.002
LVEF (%)	60.0 (59.0, 65.0)	59.0 (58.0, 61.0)	0.002
E/A ratio	0.91 (0.78, 1.13)	0.86 (0.76, 1.13)	0.168
E/e' ratio	7.9 (6.3, 9.8)	8.0 (6.2, 10.6)	0.453

*LAVI, left atrial volume index; LVMI, left ventricular mass index; LVEF, left ventricular ejection fraction; E, the peak early transmitral flow velocity; A, the peak late transmitral flow velocity; e', the lateral mitral annular velocity*.

### Cardiac Strains and LA-LV–Arterial Coupling Parameters

Left arterial phasic function was significantly reduced in non-dippers than in dippers. LA_S−S_, LA_S−E_, and LA_S−A_ were significantly lower in non-dippers (26.84 ± 6.36, 12.49 ± 4.73, and 14.30 ± 3.46%, respectively) than those in dippers (29.26 ± 6.68, 13.71 ± 4.94, and 15.24 ± 3.89%, respectively) (*P* < 0.05 for all). The LASI was significantly higher in non-dippers [0.29 (0.21, 0.41)] than in dippers [0.26 (0.21, 0.33)] (*P* < 0.05). The GLS was significantly lower in non-dippers (−18.03 ± 3.52) than in dippers (−19.17 ± 3.01) (*P* < 0.05). The difference of PWV was not statistically significant between both groups (*P* > 0.05); however, the PWV/GLS ratio was significantly higher in non-dippers [−80.9 (−69.3, −101.5)] than in dippers [−74.2 (−60.2, −90.6)] (*P* < 0.05) (Table 4).

**Table 4:** Cardiac strains and LA-LV-arterial coupling parameters.

	**Dippers**,	**Non-dippers**,	***P***
	***n* = 111**	***n* = 179**	
LA_S−S_ (%)	29.3 ± 6.7	26.8 ± 6.4	0.002
LA_S−E_ (%)	13.7 ± 4.9	12.5 ± 4.7	0.038
LA_S−A_ (%)	15.2 ± 3.9	14.3 ± 3.5	0.034
LASI	0.26 (0.21, 0.33)	0.29 (0.21, 0.41)	0.033
GLS (%)	−19.2 ± 3.0	−18.0 ± 3.5	0.005
PWV (cm/s)	1454.8 ± 368.6	1516.8 ± 311.0	0.184
PWV/GLS	−74.2 (−60.2, −90.6)	−80.9 (−69.3, −101.5)	0.029

*LAs-s, peak left atrial longitudinal strain; LA_S−E_, left atrial longitudinal strain during early diastole; LA_S−A_, left atrial longitudinal strain during late diastole; LASI, left atrial stiffness index; GLS, global longitudinal strain; PWV, pulse wave velocity*.

### Linear Regression Analysis

Univariate linear regression analysis showed that age, the comorbidity of diabetes, hypertension duration, HbA1c, BMI, eGFR, LVEF, LVMI, n-SBP, n-DBP, the percentage of n-SBP decline, and circadian rhythm pattern were correlated with LASI. Hypertension duration, LVEF, LVMI, n-SBP, n-DBP, and circadian rhythm pattern were correlated with PWV/GLS ratio (Table 5).

**Table 5:** Univariate linear regression analysis.

**LASI**	**β**	***P***	**PWV/GLS**	**β**	***P***
Age (years)	0.247	<0.001		−0.059	0.384
DM (%)	0.169	0.003		−0.102	0.130
Sex (male)	−0.003	0.958		0.079	0.241
HT duration (years)	0.235	<0.001		−0.150	0.026
BMI (kg/m^2^)	0.188	0.001		−0.086	0.204
HbA1c (%)	0.222	<0.001		−0.091	0.220
eGFR	−0.218	<0.001		0.092	0.173
LVEF	−0.267	<0.001		0.298	<0.001
LVMI	0.457	<0.001		−0.259	<0.001
n-SBP (mmHg)	0.415	<0.001		−0.264	<0.001
n-DBP (mmHg)	0.179	0.002		−0.179	0.009
Percentage of n-SBP decline (%)	−0.155	0.008		0.090	0.193
Percentage of n-DBP decline (%)	−0.083	0.159		0.078	0.256
Circadian rhythm pattern	0.130	0.027		−0.143	0.037

*DM, diabetes mellitus; HT duration, hypertension duration; BMI, body mass index; HbA1c, glycosylated haemoglobin; LVMI, left ventricular mass index; LVEF, left ventricular ejection fraction; n-SBP, nighttime mean systolic blood pressure; n-DBP, nighttime mean diastolic blood pressure*.

The associations of n-SBP, the percentage of n-SBP decline, and circadian rhythm pattern with LASI and PWV/GLS in the multivariate analysis were shown in Table 6. The associations of n-SBP and the percentage of n-SBP decline with LASI remained statistically significant in Model 1, Model 2, and Model 3. In Model 4, only n-SBP was the independent risk factor for increased LASI. The associations of n-SBP and the percentage of n-SBP decline with PWV/GLS remained statistically significant in Model 1 and Model 2. In Model 3 and Model 4, only n-SBP was the independent risk factor for increased PWV/GLS.

**Table 6:** Multivariate linear regression analysis.

**LASI**	**Model 1**		**Model 2**		**Model 3**		**Model 4**	
	**β**	***P***	**β**	***P***	**β**	***P***	**β**	***P***
n-SBP (mmHg)	0.534	<0.001	0.532	<0.001	0.510	<0.001	0.395	<0.001
Percentage of n-SBP decline (%)	0.182	0.032	0.196	0.023	0.193	0.039	0.158	0.072
Circadian rhythm pattern	0.019	0.808	0.028	0.716	0.010	0.903	−0.017	0.828
**PWV/GLS**								
n-SBP (mmHg)	−0.345	<0.001	−0.336	<0.001	−0.308	0.003	−0.247	0.024
Percentage of n-SBP decline (%)	−0.251	0.028	−0.245	0.033	−0.157	0.214	−0.134	0.288
Circadian rhythm pattern	−0.175	0.082	−0.176	0.084	−0.060	0.601	−0.045	0.688

*Model 1 was adjusted for age, sex, and BMI*.

*Model 2 was adjusted for age, sex, BMI, hypertension duration, and diabetes*.

*Model 3 was adjusted for age, sex, BMI, hypertension duration, diabetes, HbA1c, TG, TC, HDL-C, LDL-C and eGFR*.

*Model 4 was adjusted for age, sex, BMI, hypertension duration, diabetes, HbA1c, TG, TC, HDL-C, LDL-C, eGFR, LVMI and LVEF*.

*LASI, left atrial stiffness index; GLS, global longitudinal strain; PWV, pulse wave velocity; n-SBP, nighttime mean systolic blood pressure*.

## Discussion

In this study, we found that non-dippers had a significantly lower LV function (LVEF and GLS), LA phasic function (LA_S−S_, LA_S−E_ and LA_S−A_), and abnormal LA-LV–arterial coupling (LASI and PWV/GLS). Furthermore, to our knowledge, the current study for the first time, investigated the correlation between BP circadian profile and LA-LV–arterial coupling, and revealed that n-SBP rather than BP circadian rhythm, was an independent risk factor for the abnormal LA-LV–arterial coupling.

Left atrium is an active dynamic apparatus and plays a crucial role in modulating the LV filling and regulating cardiac performance integrally. In sinus rhythm, left atrium has three main phasic functions: reservoir function, collecting, and storing blood in systole; conduit function, passive passing blood to left ventricle in early diastole; booster pump function, active pumping blood to left ventricle in late diastole. 2DSTE-based strain is a sensitive and accurate parameter of LA function by evaluating LA phasic function throughout the whole cardiac cycle. LA structural and functional impairment has been regarded as the predictive biomarker of adverse cardiac events, especially for heart failure (HF) and atrial fibrillation (22, 24). Peak LA strain, known as reservoir strain, is an essential parameter of evaluating LA compliance, a sensitive predictor for LA early dysfunction (25), LV diastolic dysfunction (26), and atrial fibrillation recurrence (27). LASI is a novel measurement that combined E/e′ (the parameter reflecting LV filling pressure) and the peak LA strain (the parameter reflecting LA reservoir function). Therefore, LASI is not only an index of LA function and LA compliance, but also an indicator of LA–LV coupling. Previous studies have revealed that LASI is associated with impaired LA mechanical function (28), a marker of early target organ damage in hypertension (14), and a predictor of recurrence of atrial fibrillation (29, 30).

High blood pressure leads to a greater afterload of the heart and increases the LV stiffness (31). High blood pressure also correlates with arterial stiffness and increased PWV. The stiffening arteries lead to the early return of reflected wave, in turn increasing the late systolic LV load, which prolongs the systolic time and shortens the diastolic time, and further damages the heart (32). Hypertension is the common factor contributing to the development of HF, especially for the HF with preserved ejection fraction (HFpEF). Abnormality of LA-LV–arterial coupling is speculated to be related to development from hypertension to LV diastolic dysfunction and symptomatic HFpEF (31, 33). In this study, abnormality of LA-LV–arterial coupling evaluated by 2DSTE-based strain combined with PWV, contributed to detect subclinical hypertensive cardiovascular dysfunction.

Blunted nocturnal BP dipping is associated with adverse cardiovascular outcomes independent of BP level (9, 34). Both the BP non-dipping pattern and the nocturnal hypertension have been reported to correlate with not only LV structural and functional remodeling (6, 15, 35–37), but also LA enlargement and phasic dysfunction (16–18). In the current study, consistent with the results of previous studies, non-dipping pattern of BP was related with more severe LV hypertrophy and reduced LV systolic function. Regarding left atrium, the circadian pattern did not have significant effect on LA volume; however, the non-dippers had worse LA phasic function, including reservoir, conduit, and boost pump function. As we know, LA phasic function, particularly reservoir and conduit function, is a more sensitive indicator than LA structural remodeling to reveal LA impairment in hypertension (14, 38).

Our findings showed that the differences in 24 h and daytime BPs were not statistically significant between both groups. The variation in nighttime BPs should be the reason for the difference of LA-LV–arterial coupling between non-dippers and dippers. Furthermore, our findings revealed that nocturnal SBP level, rather than night-to-day SBP fall (non-dipping pattern), was independently correlated with the abnormality of LA-LV–arterial coupling. There were several studies showing that nighttime BP level had more impact than the circadian rhythm on cardiovascular remodeling and dysfunction. Cesare et al. (15) evaluated 1682 subjects from the PAMELA study, which was a population-based 10 years follow-up longitudinal survey in Italy, and found that nighttime BP level was the independent predictor of the development of left ventricular hypertrophy (LVH). Anne et al.'s study (37) also revealed a significant association between the n-SBP value and TOD in hypertension. Furthermore, Marijana et al.'s study (18) demonstrated that nighttime hypertension was independently correlated with the reduced LA reservoir and conduit function. The JAMP study (39), including 6,359 hypertensive patients, showed that higher nighttime BP was more important than the abnormal BP circadian pattern as a risk factor for the total cardiovascular disease. The reasons for the correlation between nighttime BP and cardiovascular risk may include two aspects, more active renin-angiotensin-aldosterone system and sympathetic nervous system, and increased circulating volume which was associated with salt sensitivity and salt intake (40–43).

Clinical implication: the LA-LV–arterial coupling was an indicator of the cardiovascular performance. The abnormality of LA-LV–arterial coupling reflected the subclinical LA and LV dysfunction, increased arterial stiffness, and furthermore provide the mechanistic between nocturnal hypertension with abnormal BP circadian profile, and the increased risks of atrial fibrillation and HF. This study demonstrated the important association between the higher n-SBP as well as non-dipping circadian pattern and the alteration of LA-LV–arterial coupling. Therefore, every hypertensive patient should be recommended to have the ABPM, in order to find nocturnal hypertension, restore the normal physiological BP circadian rhythm, and control the nighttime BP to the target level. 2DSTE-based myocardial strain and PWV should be considered to be examined for all hypertensive patients. This is very important, because of the relationship between the abnormality of LA-LV–arterial coupling and the impaired cardiovascular performance, adverse cardiovascular outcomes, such as atrial fibrillation and HF. Furthermore, we suggest further clinical studies to demonstrate the beneficial effects of controlling nighttime BP and restoring the normal physiological BP circadian rhythm on LA-LV–arterial coupling, atrial fibrillation and HF.

### Limitations

The current study had several limitations. First, antihypertensive medication may have affected haemodynamic and LA-LV–arterial coupling. Second, hypertensive patients did not record the bedtime and wake-time individually. However, all patients were advised to have a regular daily schedule in hospital. Third, we only divided patients into two groups, dippers and non-dippers, because of the limited number of patients. There were 10 extreme dippers in the group of dippers. Finally, the current study only contained the inpatient data, and further investigation is needed to extrapolate outside the hospital.

## Conclusions

The dipping pattern of BP was related to the abnormalities of myocardial mechanics and LA-LV–arterial coupling. However, an absolute n-SBP value maybe more important than BP circadian profile in the progression of abnormal LA-LV–arterial coupling.

## Data Availability Statement

The raw data supporting the conclusions of this article will be made available by the authors, without undue reservation.

## Ethics Statement

The studies involving human participants were reviewed and approved by the Ethics Committee of the First Affiliated Hospital of Dalian Medical University. The patients/participants provided their written informed consent to participate in this study. Written informed consent was obtained from the individual(s) for the publication of any potentially identifiable images or data included in this article.

## Author Contributions

QS and YZ analyzed the data. QS and YP wrote the paper. YL designed the study. YJ contributed to supervision. YL and YJ revised the manuscript. All authors have read and approved the final version of the manuscript. All authors contributed to the article and approved the submitted version.

## Conflict of Interest

The authors declare that the research was conducted in the absence of any commercial or financial relationships that could be construed as a potential conflict of interest.

## Publisher's Note

All claims expressed in this article are solely those of the authors and do not necessarily represent those of their affiliated organizations, or those of the publisher, the editors and the reviewers. Any product that may be evaluated in this article, or claim that may be made by its manufacturer, is not guaranteed or endorsed by the publisher.

## References

[1] Whelton PK Carey RM Aronow WS Casey DE Collins KJ Dennison Himmelfarb C. 2017 ACC/AHA/AAPA/ABC/ACPM/AGS/APhA/ASH/ASPC/NMA/PCNA Guideline for the prevention, detection, evaluation, and management of high blood pressure in adults: a report of the american college of cardiology/American heart association task force on clinical practice guidelines. J Am Coll Cardiol. (2018) 71:e127–248. 10.1161/HYP.0000000000000076 29146535

[2] Jones NR McCormack T Constanti M McManus RJ. Diagnosis and management of hypertension in adults: NICE guideline update 2019. Br J Gen Pract. (2020) 70:90–1. 10.3399/bjgp20X708053 32001477 PMC7018407

[3] Williams B Mancia G Spiering W Agabiti Rosei E Azizi M Burnier M. 2018 ESC/ESH Guidelines for the management of arterial hypertension. Eur Heart J. (2018) 39:3021–104. 10.1093/eurheartj/ehy339 30165516

[4] Joint Committee for Guideline R. 2018 Chinese guidelines for prevention and treatment of hypertension-a report of the revision committee of chinese guidelines for prevention and treatment of hypertension. J Geriatr Cardiol. (2019) 16:182–241. 10.11909/j.issn.1671-5411.2019.03.014 31080465 PMC6500570

[5] Abdalla M Caughey MC Tanner RM Booth JN Diaz KM Anstey DE. Associations of blood pressure dipping patterns with left ventricular mass and left ventricular hypertrophy in blacks: the jackson heart study. J Am Heart Assoc. (2017) 6:47. 10.1161/JAHA.116.004847 28381465 PMC5533000

[6] Tadic M Cuspidi C Majstorovic A Pencic B Mancia G Bombelli M. The association between 24-h blood pressure patterns and left ventricular mechanics. J Hypertens. (2020) 38:282–8. 10.1097/HJH.0000000000002241 31503137

[7] Gkaliagkousi E Anyfanti P Lazaridis A Triantafyllou A Vamvakis A Koletsos N. Clinical impact of dipping and nocturnal blood pressure patterns in newly diagnosed, never-treated patients with essential hypertension. J Am Soc Hypertens. (2018) 12:850–7. 10.1016/j.jash.2018.08.004 30219649

[8] Kario K Kanegae H Tomitani N Okawara Y Fujiwara T Yano Y. Nighttime blood pressure measured by home blood pressure monitoring as an independent predictor of cardiovascular events in general practice. Hypertension. (2019) 73:1240–8. 10.1161/HYPERTENSIONAHA.118.12740 31006331 PMC6510323

[9] Salles GF Reboldi G Fagard RH Cardoso CR Pierdomenico SD Verdecchia P. Prognostic effect of the nocturnal blood pressure fall in hypertensive patients: the ambulatory blood pressure collaboration in patients with hypertension (ABC-H) Meta-analysis. Hypertension. (2016) 67:693–700. 10.1161/HYPERTENSIONAHA.115.06981 26902495

[10] Clement DL De Buyzere ML De Bacquer DA de Leeuw PW Duprez DA Fagard RH. Prognostic value of ambulatory blood-pressure recordings in patients with treated hypertension. N Engl J Med. (2003) 348:2407–15. 10.1056/NEJMoa022273 12802026

[11] Li J Cao Y Liu C Li J Yao F Dong Y. Nocturnal systolic hypertension is a risk factor for cardiac damage in the untreated masked hypertensive patients. J Clin Hypertens (Greenwich). (2019) 21:1666–74. 10.1111/jch.13711 31556221 PMC8030335

[12] Yi JE Shin J Ihm SH Kim JH Park S Kim KI. Not non-dipping but nocturnal blood pressure predicts left ventricular hypertrophy in the essential hypertensive patients: the Korean Ambulatory Blood Pressure multicenter observational study. J Hypertens. (2014) 32:1999–2004. 10.1097/HJH.0000000000000272 25023153

[13] Little WC Pu M. Left ventricular-arterial coupling. J Am Soc Echocardiogr. (2009) 22:1246–8. 10.1016/j.echo.2009.09.023 19883874

[14] Zhao Y Sun Q Han J Lu Y Zhang Y Song W. Left atrial stiffness index as a marker of early target organ damage in hypertension. Hypertens Res. (2021) 44:299–309. 10.1038/s41440-020-00551-8 32917967

[15] Cuspidi C Facchetti R Bombelli M Sala C Negri F Grassi G. Nighttime blood pressure and new-onset left ventricular hypertrophy: findings from the Pamela population. Hypertension. (2013) 62:78–84. 10.1161/HYPERTENSIONAHA.111.00682 23690347

[16] Tadic M Cuspidi C Pencic B Mancia G Grassi G Kocijancic V. Impact of different dipping patterns on left atrial function in hypertension. J Hypertens. (2020) 38:2245–51. 10.1097/HJH.0000000000002542 32649632

[17] Demir M Aktas I Yildirim A. Left atrial mechanical function and stiffness in patients with non-dipper hypertension: a speckle tracking study. Clin Exp Hypertens. (2017) 39:319–24. 10.1080/10641963.2016.1246566 28513231

[18] Tadic M Cuspidi C Pencic-Popovic B Celic V Mancia G. The relationship between nighttime hypertension and left atrial function. J Clin Hypertens (Greenwich). (2017) 19:1096–104. 10.1111/jch.13066 28776931 PMC8030934

[19] Scuteri A Rovella V Alunni Fegatelli D Tesauro M Gabriele M Di Daniele N. An operational definition of SHATS (Systemic Hemodynamic Atherosclerotic Syndrome): role of arterial stiffness and blood pressure variability in elderly hypertensive subjects. Int J Cardiol. (2018) 263:132–7. 10.1016/j.ijcard.2018.03.117 29754908

[20] Syrseloudis D Tsioufis C Andrikou I Mazaraki A Thomopoulos C Mihas C. Association of nighttime hypertension with central arterial stiffness and urinary albumin excretion in dipper hypertensive subjects. Hypertens Res. (2011) 34:120–5. 10.1038/hr.2010.192 20962782

[21] Lang RM Badano LP Mor-Avi V Afilalo J Armstrong A Ernande L. Recommendations for cardiac chamber quantification by echocardiography in adults: an update from the American Society of Echocardiography and the European Association of Cardiovascular Imaging. J Am Soc Echocardiogr. (2015) 28:1–39. 10.1016/j.echo.2014.10.003 25559473

[22] Vieira MJ Teixeira R Goncalves L Gersh BJ. Left atrial mechanics: echocardiographic assessment and clinical implications. J Am Soc Echocardiogr. (2014) 27:463–78. 10.1016/j.echo.2014.01.021 24656882

[23] Lang RM Badano LP Mor-Avi V Afilalo J Armstrong A Ernande L. Recommendations for cardiac chamber quantification by echocardiography in adults: an update from the American Society of Echocardiography and the European Association of Cardiovascular Imaging. Eur Heart J Cardiovasc Imaging. (2015) 16:233–70. 10.1093/ehjci/jev014 25712077

[24] Hoit BD. Left atrial size and function: role in prognosis. J Am Coll Cardiol. (2014) 63:493–505. 10.1016/j.jacc.2013.10.055 24291276

[25] Singh A Medvedofsky D Mediratta A Balaney B Kruse E Ciszek B. Peak left atrial strain as a single measure for the non-invasive assessment of left ventricular filling pressures. Int J Cardiovasc Imaging. (2019) 35:23–32. 10.1007/s10554-018-1425-y 30062535 PMC6353699

[26] Abid L Charfeddine S Kammoun S. Relationship of left atrial global peak systolic strain with left ventricular diastolic dysfunction and brain natriuretic peptide level in end-stage renal disease patients with preserved left ventricular ejection fraction. J Echocardiogr. (2016) 14:71–8. 10.1007/s12574-016-0276-6 26861540

[27] Anagnostopoulos I Kousta M Kossyvakis C Lakka E Paraskevaidis NT Schizas N. The role of left atrial peak systolic strain in atrial fibrillation recurrence after catheter ablation. a systematic review and meta-analysis. Acta Cardiol. (2021) 21:1–9. 10.1080/00015385.2021.1965747 34412575

[28] Kalaycioglu E Gokdeniz T Aykan AC Hatem E Gursoy OM Cavusoglu G. Ambulatory arterial stiffness index is associated with impaired left atrial mechanical functions in hypertensive diabetic patients: a speckle tracking study. Anatol J Cardiol. (2015) 15:807–13. 10.5152/akd.2014.5796 25592109 PMC5336966

[29] Khurram IM Maqbool F Berger RD Marine JE Spragg DD Ashikaga H. Association between left atrial stiffness index and atrial fibrillation recurrence in patients undergoing left atrial ablation. Circ Arrhythm Electrophysiol. (2016) 9:163. 10.1161/CIRCEP.115.003163 26966287

[30] Shaikh AY Maan A Khan UA Aurigemma GP Hill JC Kane JL. Speckle echocardiographic left atrial strain and stiffness index as predictors of maintenance of sinus rhythm after cardioversion for atrial fibrillation: a prospective study. Cardiovasc Ultrasound. (2012) 10:48. 10.1186/1476-7120-10-48 23199055 PMC3583741

[31] Kawaguchi M Hay I Fetics B Kass DA. Combined ventricular systolic and arterial stiffening in patients with heart failure and preserved ejection fraction: implications for systolic and diastolic reserve limitations. Circulation. (2003) 107:714–20. 10.1161/01.CIR.0000048123.22359.A0 12578874

[32] Kuznetsova T D'Hooge J Kloch-Badelek M Sakiewicz W Thijs L Staessen JA. Impact of hypertension on ventricular-arterial coupling and regional myocardial work at rest and during isometric exercise. J Am Soc Echocardiogr. (2012) 25:882–90. 10.1016/j.echo.2012.04.018 22622108 PMC3607228

[33] Miyoshi H Oishi Y Mizuguchi Y Iuchi A Nagase N Ara N. Influence of comorbid cardiovascular risk factors on left atrial-left ventricular interaction in asymptomatic patients: clinical application of two-dimensional speckle-tracking echocardiography. Int Heart J. (2014) 55:138–45. 10.1536/ihj.13-220 24632964

[34] Sega R Facchetti R Bombelli M Cesana G Corrao G Grassi G. Prognostic value of ambulatory and home blood pressures compared with office blood pressure in the general population: follow-up results from the Pressioni Arteriose Monitorate e Loro Associazioni (PAMELA) study. Circulation. (2005) 111:1777–83. 10.1161/01.CIR.0000160923.04524.5B 15809377

[35] Tigen K Karaahmet T Fotbolcu H Gurel E Cevik C Gecmen C. The influence of dipper and non-dipper blood pressure patterns on left ventricular functions in hypertensive patients: a tissue Doppler study. Turk Kardiyol Dern Ars. (2009) 37:101–6. 19404031

[36] Rodrigues JCL Amadu AM Ghosh Dastidar A Harries I Burchell AE Ratcliffe LEK. Noctural dipping status and left ventricular hypertrophy: A cardiac magnetic resonance imaging study. J Clin Hypertens (Greenwich). (2018) 20:784–93. 10.1111/jch.13235 29517128 PMC8030898

[37] O'Flynn AM Dolan E Curtin RJ O'Brien E Perry IJ Kearney PM. Night-time blood pressure and target organ damage: a comparative analysis of absolute blood pressure and dipping status. J Hypertens. (2015) 33:2257–64. 10.1097/HJH.0000000000000690 26425836

[38] Li L Chen X Yin G Yan W Cui C Cheng H. Early detection of left atrial dysfunction assessed by CMR feature tracking in hypertensive patients. Eur Radiol. (2020) 30:702–11. 10.1007/s00330-019-06397-0 31515621

[39] Kario K Hoshide S Mizuno H Kabutoya T Nishizawa M Yoshida T. Nighttime blood pressure phenotype and cardiovascular prognosis: practitioner-based nationwide JAMP study. Circulation. (2020) 142:1810–20. 10.1161/CIRCULATIONAHA.120.049730 33131317 PMC7643792

[40] Kario K Williams B. Nocturnal hypertension and heart failure: mechanisms, evidence, and new treatments. Hypertension. (2021) 78:564–77. 10.1161/HYPERTENSIONAHA.121.17440 34225469

[41] Cuspidi C Sala C Tadic M Gherbesi E De Giorgi A Grassi G. Clinical and prognostic significance of a reverse dipping pattern on ambulatory monitoring: An updated review. J Clin Hypertens (Greenwich). (2017) 19:713–21. 10.1111/jch.13023 28692165 PMC8031119

[42] Satoh M Hosaka M Asayama K Kikuya M Inoue R Metoki H. Aldosterone-to-renin ratio and nocturnal blood pressure decline assessed by self-measurement of blood pressure at home: the Ohasama Study. Clin Exp Hypertens. (2014) 36:108–14. 10.3109/10641963.2014.892121 24625338

[43] Afsar B Elsurer R Kirkpantur A Kanbay M. Urinary sodium excretion and ambulatory blood pressure findings in patients with hypertension. J Clin Hypertens (Greenwich). (2015) 17:200–6. 10.1111/jch.12464 25557001 PMC8031588

